# IL-31 Associated with Coronary Artery Lesion Formation in Kawasaki Disease

**DOI:** 10.1371/journal.pone.0105195

**Published:** 2014-08-14

**Authors:** Wan-Ning Tseng, Mao-Hung Lo, Mindy Ming-Huey Guo, Kai-Sheng Hsieh, Wei-Chiao Chang, Ho-Chang Kuo

**Affiliations:** 1 Department of Pediatric, Kaohsiung Chang Gung Memorial Hospital, Chang Gung University College of Medicine, Kaohsiung, Taiwan; 2 Kawasaki Disease Center, Kaohsiung Chang Gung Memorial Hospital, Kaohsiung, Taiwan; 3 Department of Clinical Pharmacy, School of Pharmacy, Taipei Medical University, Taipei, Taiwan; National Central University, Taiwan

## Abstract

**Background:**

Kawasaki disease (KD) is known to be associated with T help (Th) 2 reaction and subsequently allergic diseases. Interleukin-31 (IL-31) has also been reported to be involved in Th2 mediated diseases such as allergic diseases. However, the role of IL-31 in KD has not been previously reported. The aim of this study is to investigate whether IL-31 is associated with KD and its clinical outcome.

**Material:**

A total of 78 KD patients who met the criteria of KD were enrolled in this study as well as 20 age-matched controls. Plasma samples were conducted to measure IL-31 before intravenous immunoglobulin (IVIG) treatment (KD1), within 3 days after IVIG treatment (KD2) and at least 3 weeks after IVIG treatment (KD3) by utilizing enzyme-linked immunosorbent assay (ELISA).

**Result:**

Our findings showed that IL-31 expression was higher in KD patients after IVIG treatment significantly (KD2>KD1: 1265.0±199.3 vs. 840.2±152.5 pg/ml, p<0.0001). Further analysis revealed that IL-31 level was significantly higher in KD patients with coronary artery lesion (CAL) (656.6±139.5 vs. 1373.0±422.0 pg/ml, p = 0.04) before IVIG treatment (KD1). There were no significant differences between the IVIG resistance and IVIG responsiveness groups.

**Conclusion:**

IL-31 was increased after IVIG treatment in patients with KD and was significantly associated with CAL formation. The results from this study may help to identify a novel risk factor for predicting KD and CAL formation.

## Introduction

Kawasaki disease (KD) is an acute multisystemic vasculitis with fever of unknown causes, which was first found by Kawasaki et al. [1] It is reported worldwide in all populations, with the highest incidence among children less than 5 years old especial with an Asian background. The most serious complication of KD is coronary artery lesions (CAL), including aneurysms formation or coronary artery dilatation. Approximately 20∼25% of untreated with intravenous immunoglobulin (IVIG) patients experience coronary artery abnormalities. The suggested global standard treatment for patients with acute KD is a high dose (2 g/kg) of IVIG in single injection and aspirin.

Interleukin-31 (IL-31) is a IL-6 family cytokine that is expressed in kinds of human tissues [2] and at relatively high levels by activated CD4+ T cells, especially cells skewed toward a T help (Th) 2-phenotype [3]. IL-31 binds to a heterodimeric receptor, consisting of the IL-31 R alpha (IL-31 RA) and oncostatin M receptor beta (OSMR) that is constitutively expressed on epithelial cells. Ulrike et al. found that increased levels of IL-31 were associated with Th2 cytokines including IL-4 and IL-13 in children with atopic dermatitis [4]. Moreover, IL-31 was found to be associated with asthma and allergic rhinitis [5], [6].

Th2 immune response included IL-4 [7], IL-5, eosinophil [8] and CCL17 [9] were also reported to play some role in the immuneopathogenesis and outcome of KD [10]. Eosinophilia associated with KD was first described by Dr. Kawasaki et al. [1] and was also found in coronary artery autopsies [11]. Previously, we found that peripheral eosinophilia was related to IVIG therapy response rate [8]. An increase in eosinophil was also found in patients with enterovirus (EV) infection after being treated with IVIG, but there was not as much of an increase as patients with KD after IVIG treatment. The increase of eosinophilia may be related to IVIG therapy in KD and/or EV patients. KD patients had higher eosinophil levels both before and after IVIG therapy as compared with EV infection patients, which may due to the nature disease course or inflammatory mechanism of KD [12].

Prevalence of atopic dermatitis, asthma and allergic rhinitis were reported to be higher in KD-affected children from a population-based study in Taiwan [13]–[15]. The related data suggests that children with history of KD were at a higher potential of developing allergic diseases. Blood IL-31 has been correlated to disease severity in atopic dermatitis [16] but had not been studied in KD to date. This study was performed to assess whether IL-31 plays a role in patients with KD, and to examine the association of IVIG treatment response and CAL formation.

## Materials and Methods

### Patients

All subjects studied were children who fit the criteria of KD [17] and received IVIG treatment after admission to the Kaohsiung Chang Gung Memorial Hospital from 2008 to 2012. KD patients were treated with single dose of IVIG (2 g/kg) administered over 8–12 hour period of time. Low dose aspirin (3∼5 mg/kg/day) was administrated until all inflammation signs were resolved or until regression of CAL. This study was approved by the Institutional Review Board (IRB) of the Chang Gung Memorial Hospital. The IRB approved this consensual procedure (98–3674B). Blood samples were collected after written informed consent was obtained from parents or guardians. The participant’s consent was recorded utilizing a decoded method. Blood samples collected before (before IVIG treatment, KD1) and after IVIG treatment (within 3 days, KD2; at least 3 weeks after IVIG, KD3) were subjected to this study. Patients whose symptoms did not match the AHA diagnostic criteria of KD (fever more than 5 days), or those involved in the incomplete collection of pre- and post-IVIG blood samples were excluded. CAL were defined as the internal diameter of coronary artery great than 3 mm (age less than 5 yr), or greater than 4 mm (age greater than 5 yr) or the internal diameter of a segment at least 1.5 times than that of an adjacent segment as previously reported [18], [19]. Blood samples from febrile control (FC) group included for comparison were patients admitted for upper and/or lower respiratory tract infections (including acute pharyngitis, enterovirus, acute bronchiolitis, acute bronchitis, croup, and acute tonsillitis) without any history of KD and allergic diseases. Plasma levels of IL-31 were detected by enzyme-linked immunosorbent assay (ELISA, Duoset ELISA Development Systems, R&D Systems).

### Statistical analysis

Changes of plasma IL-31 levels before and after IVIG treatment were analyzed by paired sample *t*-test. Plasma levels of IL-31 between KD patients with and without CAL and correlations between groups were tested by student *t* test. A p-value of <0.05 was considered as being statistically significant. All statistical tests were performed using SPSS 14.0 for Windows (SPSS, Inc., Chicago, IL, USA).

## Results

A total of 78 KD patients whose blood samples collected both before and after IVIG treatment were enrolled in this case-control study. The major clinical phenotypes of KD patients including non-purulent conjunctivitis, fissured lips, strawberry tongue, polymorphous skin rashes, indurations of extremities, cervical lymphadenopathy, and erythematous change at the bacillus Calmette-Guerin (BCG) vaccination scar were recorded and showed in Table 1 and Table 2. There were 52 boys (66%) and 27 girls (34%). As shown in Table 1, there were 20 patients (25.31%) with CAL. The age distribution between KD and control patients showed no significant difference (1.62±1.51 vs. 1.62±0.42 year-old, p = 0.98). The age distribution of KD patients with or without CAL and IVIG responsiveness or IVIG resistance also showed no significant difference (p>0.05). As seen in Table 2, there was no significant difference in clinical manifestations between high and low plasma levels of IL-31 group that was divided by cut point of IL-31 medium value (p>0.05).

**Table 1:** Demographic data of Kawasaki disease patients and the control group.

	KD	FC	P value
	(n = 78)	(n = 20)	
Age (years)	1.62±1.51	1.62±0.42	0.98
Male gender (%)	52 (66.0%)	7 (34.0%)	0.01
CAL formation, no. (%)	20 (25.31%)		
IVIG resistant, no. (%)	8 (10.13%)		

Data was showed with mean and standard deviation.

KD: Kawasaki disease; FC: Fever Control; CAL: coronary artery lesion; IVIG: intravenous immunoglobulin.

**Table 2:** Comparison of clinical symptoms in low vs. high plasma levels of IL-31.

IL-31	IL-31 Low	IL-31 high	P value
	<290.94 pg/ml	≧290.94 pg/ml	
Cervical lymphadenopathy (%)	40.0%	60.0%	0.35
Fissured lips or strawberry tongue (%)	50.0%	50.0%	0.33
Polymorphous skin rashes (%)	49.3%	50.7%	0.98
Non-purulent conjunctivitis (%)	50.7%	49.3%	0.31
Indurational of extremities (%)	50.9%	49.1%	0.95

High and low group was divided by medium value of plasma IL-31 level.

### Plasma levels of IL-31 increase in KD patients

**Figure 1:**
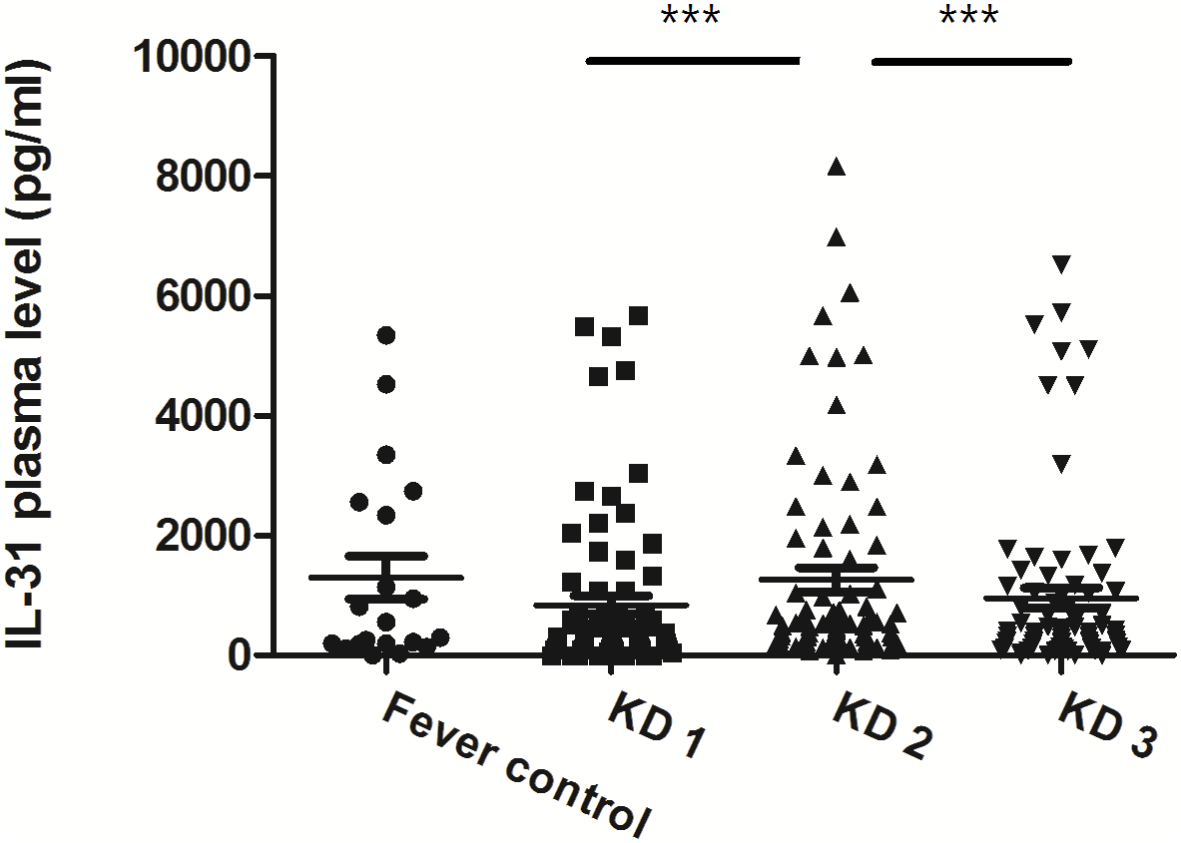
IL-31 was significantly higher in KD2 (within 3 days after IVIG treatment) when compared with KD1 (before IVIG treatment) and KD3 (at least 3 weeks after IVIG treatment). ***P<0.0005. P values were tested by Paired sample t-test.

As shown in figure 1, we found that IL-31 was significantly higher in KD patients after IVIG treatment (KD1 vs. KD2: 840.2±152.5 vs. 1265.0±199.3 pg/ml, p<0.0001) when compared with before IVIG treatment (KD1). Plasma levels of IL-31 in KD3 were significantly decreased to the same range as KD1 and controls (KD2 vs. KD3: 1265.0±199.3 vs. 956.7±168.5 pg/ml, p<0.0001). There is no significant difference between FC (1296±360.9 pg/ml) and KD1 (p>0.05).

### IL-31 higher in KD with CAL formation

**Figure 2:**
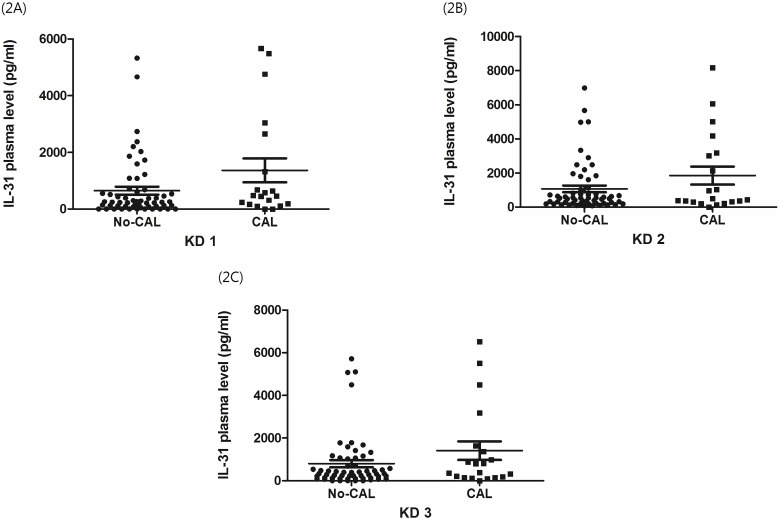
Increase of plasma interleukin-31 levels associated with coronary artery lesion formation in Kawasaki disease. Distribution of plasma levels of interleukin-31 (IL-31) before Intravenous immunoglobulin (IVIG) treatment (KD1), within 3 days after IVIG treatment (KD2) and at least 3 weeks after IVIG treatment (KD3). The display (2A) showed significant higher levels of IL-31 in Kawasaki disease patients with CAL formation before IVIG treatment (656.6±139.5 vs. 1373.0±422.0 pg/ml, p = 0.04) when compared with without CAL formation. After IVIG treatment, plasma levels of IL-31 showed no significant difference between patients with or without CAL (2B and 2C).

Before IVIG treatment, higher IL-31 levels were observed and were shown to have a statistical significance in patients with CAL (656.6±139.5 vs. 1373.0±422.0 pg/ml, p = 0.04) when compared with KD patients without CAL formation. After IVIG treatment, IL-31 was still higher in KD patients with CAL but this did not reach a statistically significant difference (KD2: 1064.0±194.3 vs. 1847.0±524.4 pg/ml, p = 0.09; KD3: 800.8±168.0 vs. 1409.0±434.5 pg/ml, p = 0.12) (as shown in Figure 2, p>0.05).

**Figure 3:**
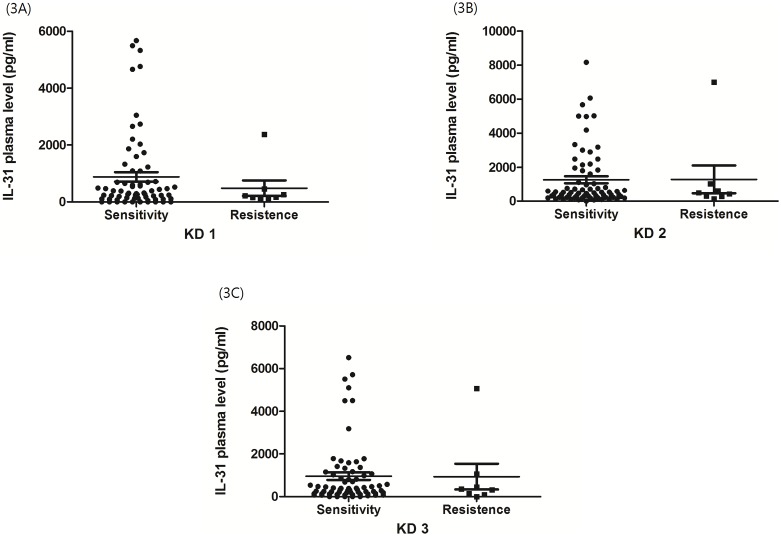
IL-31 levels in KD patients showed no significant association with IVIG resistance. The display (3A) showed no significant difference (880.9±166.7 vs. 484.4±273.0 pg/ml, p = 0.43) in patients before IVIG treatment. Data also showed no significant difference in KD2 (1263.0±204.0 vs. 1285.0±818.9 pg/ml, p = 0.97) (3B) and KD3 (958.7±176.4 vs. 938.8±602.0 pg/ml, p = 0.97) (3C).

In order to determine whether IL-31 level is related to IVIG treatment response, we compared the plasma levels of IL-31 from patients with IVIG responsiveness and resistance. In those who received IVIG treatment once due to persistent fever or inflammatory sign after the initial IVIG treatment, 48 hours were considered as being representative of IVIG resistance. Plasma levels of IL-31 showed no significant difference in KD1 (880.9±166.7 vs. 484.4±273.0 pg/ml, p = 0.43) as well as in KD2 (1263.0±204.0 vs. 1285.0±818.9 pg/ml, p = 0.97) and KD3 (958.7±176.4 vs. 938.8±602.0 pg/ml, p = 0.97) (as shown in Figure 3, p>0.05).

## Discussion

Kawasaki disease is a systemic vasculitis and mainly caused coronary artery lesions which is the most common cause of acquired heart disease in children. Previous studies have shown that the immune system including Th1 immune related response such as interferon-Gamma, tumor necrosis factor-alpha, IL-1 and IL-10 as well as Th2 immune related response such as IL-4, IL5 and IL-13 were highly expressed during the acute stage of KD [20], [21].

IL-31 is known to associate with Th2 cytokines (IL-4, IL-5, and IL-13) and a possible interaction between IL-31 and Th2 inflammation has been suggested. For the first time, our present study provides evidence that IL-31 expression is closely related to Kawasaki disease, and that it may also be a predictor of CAL formation. A number of clinical and laboratory factors including young age, male gender, prolonged fever, initially high C-reactive protein (CRP) and higher neutrophil counts have been implicated in the prediction of CAL [22]–[24], but the mechanism remains unclear. Several studies have shown the role of leukocyte counts, especially eosinophils, as a risk factor for patients with coronary artery disease. Terai et al. reported that an accumulation of eosinophils in the coronary micro-vessel lesions and eosinophilia in peripheral blood of KD vasculitis [11]. Prentice et al. [25] and Hospers et al. [26] have reported a relationship between eosinophil and coronary artery diseases. Eosinophils are involved in inflammatory reactions and direct activities eosinophil-derived mediators such as leukotriene C4 and D4, histamine, and prostaglandin D2, from mast cells and basophils. Such vasoactive substances will cause smooth muscle contraction. Cheung et al. pointed out that IL-31 could significantly stimulate the release of pro-inflammatory cytokines from eosinophils, via functional cell surface IL-31 receptor [27]. This may be the mechanism that contributes to elevated IL-31 in CAL patients of KD. In our study, IL-31 is much higher in those who develop CAL, but the disparity of IL-31 before and after treatment showed no significant difference. This suggests that high IL-31 levels before IVIG therapy may be a risk factor to be considered in the prediction of CAL.

In our previous study, we have shown that eosinophilia after IVIG treatment had an inverse association with with IVIG resistance in KD patients [8]. Kobayashi at el. demonstrated that KD patients who failed to respond to the first dose of IVIG treatment had certain risk factors including serum sodium concentration less than 133 mmol/L, less than 4 days illness at diagnosis, elevated aspartate aminotransferase concentration more than 100 U/L, neutrophils greater than 80%, platelet count less than 30×10^4^/µL, CRP concentration more than 100 mg/L and age younger than 12 months [28]. In our study, either IL-31 level or disparities of IL-31 levels (difference between KD1, KD2 and KD3) are not associated with IVIG resistance. This may be that absolute eosinophil count or change of absolute eosinophil count did not positively correlate with disparities of IL-31 in KD patients. Thus, IL-31 does not play a role in predicting IVIG resistance.

Matsuoka et al. reported a cross-sectional survey that allergic rhinitis and/or atopic dermatitis were more common in children with KD history as compared with non-KD history controls [14]. Webster et al. found that KD patients were more likely to be admitted to hospital due to asthma and/or allergic disease than non-KD patients [29]. Taking together, there results suggest that KD patients are at an increased risk of developing allergic diseases. The mechanism by which KD may increase the risk of further allergic disease remains unclear, but eosinophilia and abnormal Th1/Th2 balance in the acute stage of KD may be associated. IL-31 can directly induce Th2 cytokine such as IL-4, IL-5 and IL-13 production [30]. These results suggest that IL-31 induced a higher expression of Th2 cytokines, which induced more severe Th2 inflammation and aggravating clinical symptoms. Our findings showed that IL-31 expression is higher in KD patients after IVIG treatment and may be the cause of increases in allergic diseases. Whether this result is associated with the disease or IVIG therapy remains unclear, but it does provide us with a topic for further study in terms of KD and allergic diseases.

## Conclusions

In conclusion, this study is the first study to investigate the relationship of IL-31 and KD. Our study showed positive evidence that IL-31 was highly increased in KD patients who develop CAL. This may provide evidence of a new risk factor that may be used when predicting KD with CAL and it also provides us with data that can be utilized when examining whether or not these patients are also at risk of developing allergic diseases.
